# The Medical Council and the Royal Colleges

**Published:** 1900-12-22

**Authors:** Thomas Bryant

**Affiliations:** 65 Grosvenor Street, Grosvenor Square


### THE MEDICAL COUNCIL AND THE ROYAL COLLEGES.

To the Editors of *The Hospital.*

Dear Mr. Editor,—I well know you wish to express what is true in your paper, and consequently was surprised to find you following a cry from prejudiced bodies against the Royal Colleges which is so far from the truth as to be misleading. Please read again my memorandum, when I suppose you will admit that the Royal Colleges have taken much trouble to visit the places accepted and have only accepted what are clearly first-class; at any rate, they are not such places as you would lead your readers to believe.

Yours faithfully,
Thomas Bryant.

65 Grosvenor Street, Grosvenor Square,
December 16, 1900.

[We print Mr. Bryant's letter, but do not recognise the existence of any ground for his complaint. We quoted his own words in explanation of the way in which the Royal Colleges had been induced to depart from their original intentions with regard to the study of chemistry, physics, and biology. In speaking of "any school which the authorities of any licensing body may choose to consider good enough for recognition," we neither said nor implied that such recognition had been granted, so far, without consideration and inquiry. Mr. Bryant's statement that the Royal Colleges which he represents "have taken the trouble to visit the places accepted, and have only accepted what are clearly first-class" is interesting and important, but it has nothing to do with our comments, which refer, as he will see if he reads our article again, to the "other colleges and universities" of which he speaks in the memorandum on which we commented, colleges over the doing of which his college can exercise no control whatever. It is Mr. Bryant, not we, who says that the London colleges have "adopted the ways" of these "other colleges." The question at issue is one of principle, and if the control of the General Medical Council were removed, the inevitable tendency among a number of competing colleges and universities in different parts of the country would be towards declension from whatever standard might in the first instance be adopted.]

—Ed. The Hospital.

